# Oxygen Evolution at Manganite Perovskite Ruddlesden-Popper Type Particles: Trends of Activity on Structure, Valence and Covalence

**DOI:** 10.3390/ma9110921

**Published:** 2016-11-14

**Authors:** Majid Ebrahimizadeh Abrishami, Marcel Risch, Julius Scholz, Vladimir Roddatis, Norbert Osterthun, Christian Jooss

**Affiliations:** Institute of Materials Physics, University of Goettingen, Friedrich-Hund-Platz 1, Goettingen 37077, Germany; majid@material.physik.uni-goettingen.de (M.E.A.); mrisch@material.physik.uni-goettingen.de (M.R.); jscholz@material.physik.uni-goettingen.de (J.S.); vroddatis@material.physik.uni-goettingen.de (V.R.); nosterthun@material.physik.uni-goettingen.de (N.O.)

**Keywords:** oxygen evolution reaction, manganite perovskite, Ruddlesden-Popper systems, electrocatalyst, X-ray absorption spectroscopy

## Abstract

An improved understanding of the correlation between the electronic properties of Mn-O bonds, activity and stability of electro-catalysts for the oxygen evolution reaction (OER) is of great importance for an improved catalyst design. Here, an in-depth study of the relation between lattice structure, electronic properties and catalyst performance of the perovskite Ca_1−x_Pr_x_MnO_3_ and the first-order RP-system Ca_2−x_Pr_x_MnO_4_ at doping levels of x = 0, 0.25 and 0.5 is presented. Lattice structure is determined by X-ray powder diffraction and Rietveld refinement. X-ray absorption spectroscopy of Mn-L and O-K edges gives access to Mn valence and covalency of the Mn-O bond. Oxygen evolution activity and stability is measured by rotating ring disc electrode studies. We demonstrate that the highest activity and stability coincidences for systems with a Mn-valence state of +3.7, though also requiring that the covalency of the Mn-O bond has a relative minimum. This observation points to an oxygen evolution mechanism with high redox activity of Mn. Covalency should be large enough for facile electron transfer from adsorbed oxygen species to the MnO_6_ network; however, it should not be hampered by oxidation of the lattice oxygen, which might cause a crossover to material degradation. Since valence and covalency changes are not entirely independent, the introduction of the energy position of the *e_g_*^↑^ pre-edge peak in the O-K spectra as a new descriptor for oxygen evolution is suggested, leading to a volcano-like representation of the OER activity.

## 1. Introduction

The oxygen evolution reaction (OER) is the bottleneck in electro-chemical water splitting. Transforming two H_2_O molecules into molecular O_2_ via a four-step electron transfer reaction is demanding and requires large overpotentials. Understanding the mechanism of this reaction at different electrocatalysts is consequently at the center of the current research and essential for the design of highly efficient catalysts from abundant materials [1,2,3,4,5,6,7,8]. Metal oxides are a natural choice for catalysts [9,10,11,12,13,14,15,16,17] because even noble metals such as Pt form surface oxides under the highly oxidizing conditions of the OER [18]. Many binary metal oxides are insulators [19], either due to the ionic nature of the metal-oxygen bond or electronic correlations. Such materials are not suitable for the catalysis of electron transfer reactions because of poor electrical conductivity. However, in some transition metal oxides, particularly perovskites, suitable hybridization of transition metal *d* and oxygen 2*p* states can give rise to covalent bonding contributions [19,20,21]. Based on the assumption that metals represent the catalytic active site for OER in theoretical work [22,23], material design of highly active catalysts may follow two partially contradicting requirements [24]. First, the metal center must be able to develop different oxidation states that can adapt to the different involved oxidation steps during OER which requires ionic charge. Simultaneously, the metal oxygen bond must exhibit sufficient covalency for facile electron transfer. This requires suitable hybridization and charge delocalization.

The study of perovskite oxides, ABO_3_, consisting of a rare earth or alkaline earth metal A-site and a transition metal (TM) B-site is attractive due to the thermodynamic stability of the structure. In addition, the valence state of the B-site metal in such perovskites can be modified via A-site doping through heterovalent substitution of cations. Valence changes also affect the covalency of TM-O bonds due to the changes of bond length and tilt angle that depend on the filling of the *d* states involved. Indeed, on the basis of a rigid molecular orbital argument, a simple descriptor of OER activity has been proposed by Suntivich et al. [25]; it suggests that the highest OER activity can be attained when the 3*d* *e_g_* occupancy of the B-site TM is slightly above unity. They further observed that the OER activity was enhanced with increasing covalent mixing between B-site ion and O atoms of the oxides at a constant *e_g_* filling, suggesting that a larger O-2*p* character of the conduction band of the active redox couple, i.e., the B-site, promotes the charge transfer between surface cations and oxygen-related adsorbates [20]. Based on such arguments, the systems which have been designed, e.g., Ba_0.5_Sr_0.5_Co_0.8_Fe_0.2_O_3_ (BSCF), show high OER activity. However, they suffer from surface amorphization during OER [26,27] due to oxygen vacancy formation and an unfavorable O-*p* band location [28].

**Figure 1:**
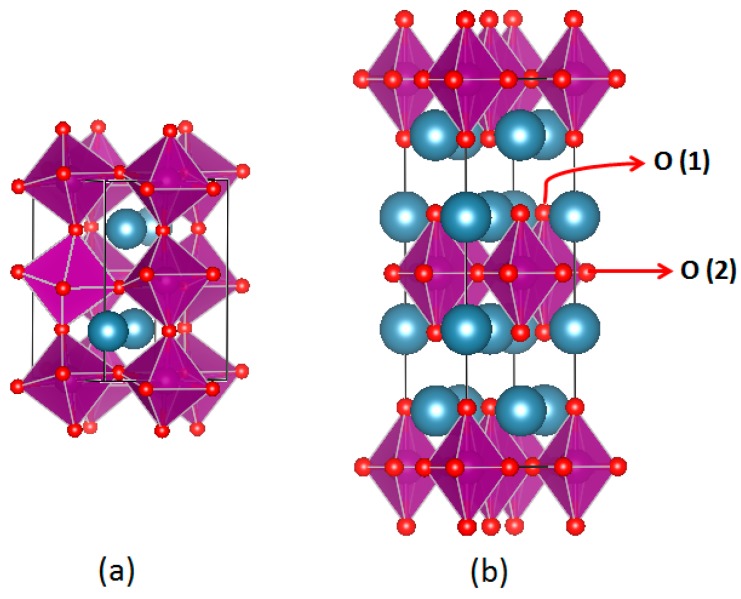
Representation of (**a**) the perovskite oxide, ABO_3_ (*n =* ∞ in the Ruddelsden-Popper system) and (**b**) the first-order (*n =* 1) Ruddlesden-Popper system A_2_BO_4_. O(1) and O(2) oxygen atoms are located at an apical site in rock salt and an equatorial site in perovskite layers, respectively.

Recently, a class of layered perovskites, known as Ruddlesden-Popper series (A_n+1_B_n_O_3n+1_), has attracted the attention of researchers, since these materials may provide interstitial oxygen diffusion [29]. Ruddlesden-Popper (RP) systems can be derived from the perovskite structure by stacking n perovskite ABO_3_ layers onto one rock-salt AO layer along the c-direction (Figure 1). Due to flexible uptake of interstitial oxygen at the tetrahedral site between the ABO_3_ and AO layers and the preferential formation of oxygen vacancies at the apical O-site pointing toward the rock-salt layers, such systems show higher structural flexibility in accommodating oxygen non-stoichiometry [30,31,32]. This suggests higher stability of such systems during OER, if catalysis is accompanied by surface formation of oxygen vacancies as recently proposed for some specific perovskites [24,33,34,35,36,37].

In this work, the relations between atomic, electronic structure, oxygen evolution activity and stability are studied for perovskites (P) Ca_1−x_Pr_x_MnO_3_ which can be expressed as an RP system with *n =* ∞ and the first-order RP-systems (*n =* 1) Ca_2−x_Pr_x_MnO_4_ at doping levels of x = 0, 0.25 and 0.5. We have selected these systems, due to similarity in chemical composition to the CaMn_4_O_5_ complex that is the oxygen evolution center in natural photosynthesis [38]. (We note that the structure and consequently the catalytic mechanism of P and RP-systems differ considerably from that of natural photosynthesis.) The accessible valence states of manganese between 2+ and 4+ provide high flexibility in the electronic structure [39], which can adapt to the oxidation steps during OER. Moreover, Siegbahn et al. [40] showed that the calcium cofactor is preferable for the formation of the six-coordinate Mn^IV^O• oxyl state, which may be an important pre-step for O_2_ formation. In solid-state systems, the possible redox activity of lattice oxygen may mimic the chemistry of such oxyl radicals to some extent [33]. Redox activity of the lattice oxygen, i.e., the formation of O^−^ states, depends on the contribution of oxygen 2*p* states to the unoccupied metal 3*d*-states, i.e., the covalency of the upper edge of valence or lower edge of conduction band of the solid. Oxidation of lattice oxygen can however not only be involved in the OER mechanism, but may represent a pathway to oxygen vacancy formation. It thus has a significant impact on the activity and stability [33,37]. Oxygen defects including vacancies and interstitials in perovskite oxides change the surface atomic structure and consequently affect Mn-O bond strength, besides the possibility of inducing catalyst corrosion [24,36,37].

The work presented in this article is based on single-phase nanoparticles of the perovskite Ca_1−x_Pr_x_MnO_3_ (RP system with *n =* ∞) and the first-order RP structure (*n =* 1) Ca_2−x_Pr_x_MnO_4_, both with the A-site doping levels of x = 0.00, 0.25, 0.50. The crystal structure was determined by X-ray diffraction and Rietveld refinement, giving access to the doping-dependent change of Mn-O bond length and bond angles. High-resolution TEM images and electron diffraction show that particles are single crystalline and defect poor. Particle size and size distribution functions were determined by SEM and statistical analysis. This was the basis for careful electro-chemical analysis of the activity and stability of the particles during OER under alkaline conditions. We show how activity and stability depend on the Mn valence state as well as the covalency, indicating that maximum activity at a valence of Mn^3.7+^ can be only maintained if the high covalency of the Mn *e_g_*–O 2*p* antibonding states in the perovskite is reduced. Thus maximizing Mn redox activity and reducing the 2*p* character of charge carriers while maintaining facile electron transfer may be a pathway of design for stable and active abundant manganite perovskite catalysts. By correlating trends in structure refinement and X-ray spectroscopy of the O-K edge, we suggest that this can be achieved in the RP phases due to the small octahedral tilt that enables facile electron transfer, and the pronounced lifting of the degeneracy of the Mn *e_g_* states by the layered RP structure that reduces O 2*p* contributions to the conduction band. Based on the observed trends, and since valence and covalency changes are not independent, we suggest introducing a new descriptor to account for the subtler electronic effects in perovskite and layered systems.

## 2. Results and Discussion

### 2.1. Crystal Structure

**Figure 2:**
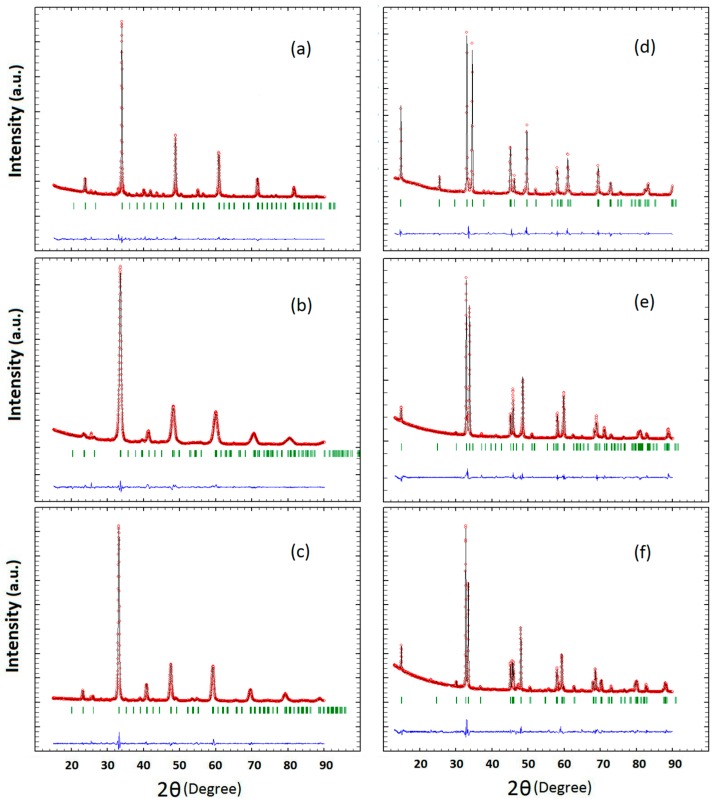
Observed (○) and calculated (solid black line) XRD patterns for perovskite oxides Ca_1−x_Pr_x_MnO_3_ series (**a**) P00; (**b**) P25 and (**c**) P50 as well as the Ruddlesden-Popper Ca_2−x_Pr_x_MnO_4_ series (**d**) RP00; (**e**) RP25 and (**f**) RP50, respectively. The expected pattern is shown in green and the residual (blue line) is plotted at the bottom of each pattern.

The XRD data of the P- and RP-series (Ca_1−x_Pr_x_MnO_3_ and Ca_2−x_Pr_x_MnO_4_ with x = 0.00, 0.25, 0.50) are shown in Figure 2a–f) and their analysis in Table 1 and Table 2. The lattice parameters and the space groups of the P-series, Pnma (no. 62), are consistent with that of earlier reports [41,42]. Although the ionic radius of Pr^3+^ (1.13 Å) is close to that of Ca^2+^ (1.12 Å) [43], the lattice constants increase with the increase of Pr content, because the Mn-O bond length increases with increasing electron occupation of the antibonding Mn-*e_g_*–O-2*p* levels. In case of the RP-series, the space group changes with the doping level. For the RP00, we found a space group of I 41/acd and the lattice parameters which are consistent with those obtained by Fawcett et al. [41]. We identified the orthorhombic space group of Fmm2 for RP50 which is not consistent with previous reports on tetragonal symmetry of this system [44,45]. However, the Fmm2 space group has been reported for Ca_2−x_Nd_x_MnO_4_ [44]. For RP25, we found the best fit for the tetragonal space group, I/4 mmm, with lattice parameters close to that reported by Daoudi et al. [44] and in contrast to the orthorhombic charge ordered structure, which was found at room temperature in reference [45]. It is quite natural that the space group of the RP-systems is changed by doping because of different ordering of the Pr and Ca atoms and induced changes of the octahedral tilt systems. Octahedral tilting results in the anion moving off of a high symmetry site to a lower symmetry site.

Mn-O bond distances and Mn-O-Mn angles obtained after refinement are given in Table 2. There are three distinct Mn-O bond distances for the P-series, one distance for Mn-O(1) with bond angles below 160° and two distances for Mn-O(2) with bond angles above 160°. In case of the layered RP-series, the distortion in MnO_6_ octahedra can be characterized by lengthening the bond distance of Mn-O(1) in apical sites at the edge of perovskite like slabs neighboring the rock-salt CaO layers [41]. Two apical oxygen distances were found in RP50. We observed a considerable lengthening of the Mn-O(1) bond to about 2 Å, while perovskite oxides with the same Pr stoichiometry have a Mn-O(2) distance of about 2 Å with the exception of P00. Furthermore, the Rietveld refinements show that increasing Pr doping causes a slight decrease in Mn-O(1) bond lengths within the RP-series, but shows no clear trend for the P series. Remarkably, the Mn-O(2)-Mn bond angle strongly increases from RP00 to the Pr-doped RP-systems. We assume that the strong octahedral tilt distortion observed in RP00 is due to oxygen-deficient structures which is very common in Ca-rich manganites [46,47] and is consistent with the valence analysis presented below. The change in the topology of the MnO_6_ network from three-dimensional corner sharing in the P system to two-dimensional corner sharing in the RP system with O(1) oxygen atoms at apical sites bonded only to one Mn atom has a strong impact on the orbital hybridization between O-2*p* and Mn-3*d* states. Oxygen atoms in apical sites can result in *t*_2*g*_ orbitals splitting to *d_xz_* and *d_yz_* that are more stabilized than *d_xy_* orbitals and consequently lead to lower energy for a *d*^3^ configuration [41].

### 2.2. Particle Size and Microstructure

**Figure 3:**
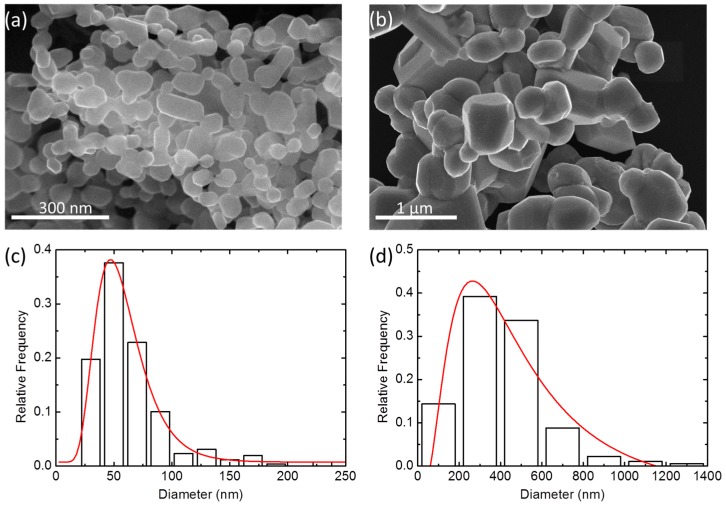
SEM images of the (**a**) perovskite (P50) and (**b**) Ruddlesden-Popper (RP50) particles. Histograms in (**c**) and (**d**) show the size distribution functions obtained for P50 and RP50, respectively.

Figure 3a,b show the morphologies and microstructures of the P50 and RP50 particles, representing typical results for the P-series and RP-series, respectively. Histograms in the bottom of each image show the size distribution of the particles. Histograms for other doping levels can be found in the supplementary material (Figure S1). The P samples prepared by the wet-chemistry method were composed of nanoparticles with an average size of 60 nm, while the average particle size of RP phase samples obtained from solid-state reaction of nanoparticles was more than 200 nm (Table S1).

**Figure 4:**
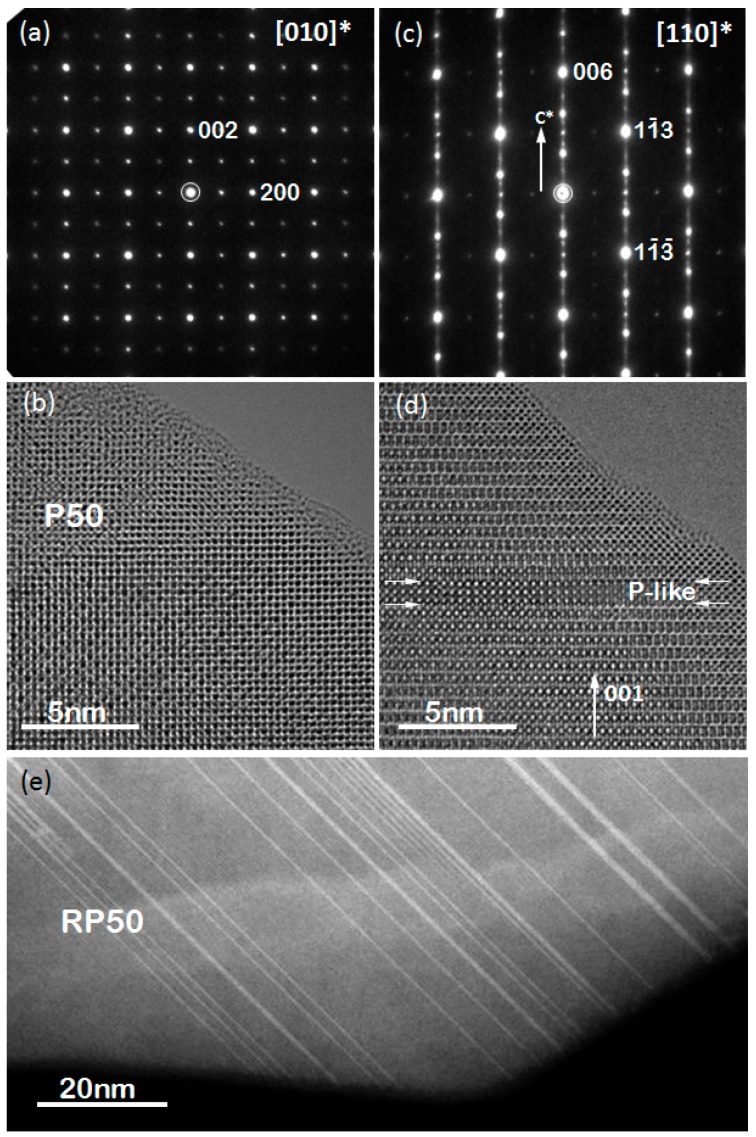
(**a**) Electron diffraction (ED) pattern of P50 along [010] and (**b**) corresponding HRTEM image; (**c**) ED pattern of RP50 along [110] and (**d**) corresponding HRTEM image. Note the appearance of diffuse streaks along [001] direction and forbidden spots in ED patterns in (**c**) caused by intergrowth of Pr-rich AMnO_3_ layers indicated by white arrows in (**d**) and visualized by HAADF STEM imaging in (**e**).

Figure 4a,c show electron diffraction (ED) patterns of P50 and RP50 along [010]* and [110]* zone axes, respectively. The diffraction patterns were completely indexed based upon the space groups Pnma (no. 62) for Ca_1−x_Pr_x_MnO_3_ (P-PCMO) and Fmm2 (no. 42) for Ca_2−x_Pr_x_MnO_4_ (RP-PCMO) and using the unit cell parameters determined by powder XRD. The presence of diffuse streak lines parallel to the [001] direction and forbidden spots in ED patterns in RP-PCMO can be explained by the numerous violations of layer sequence by individual Pr layers or even PCMO intergrowths, visible in the HRTEM image in Figure 4d and the STEM overview image in Figure 4e.

### 2.3. Electrochemical Activity and Stability Tests

**Figure 5:**
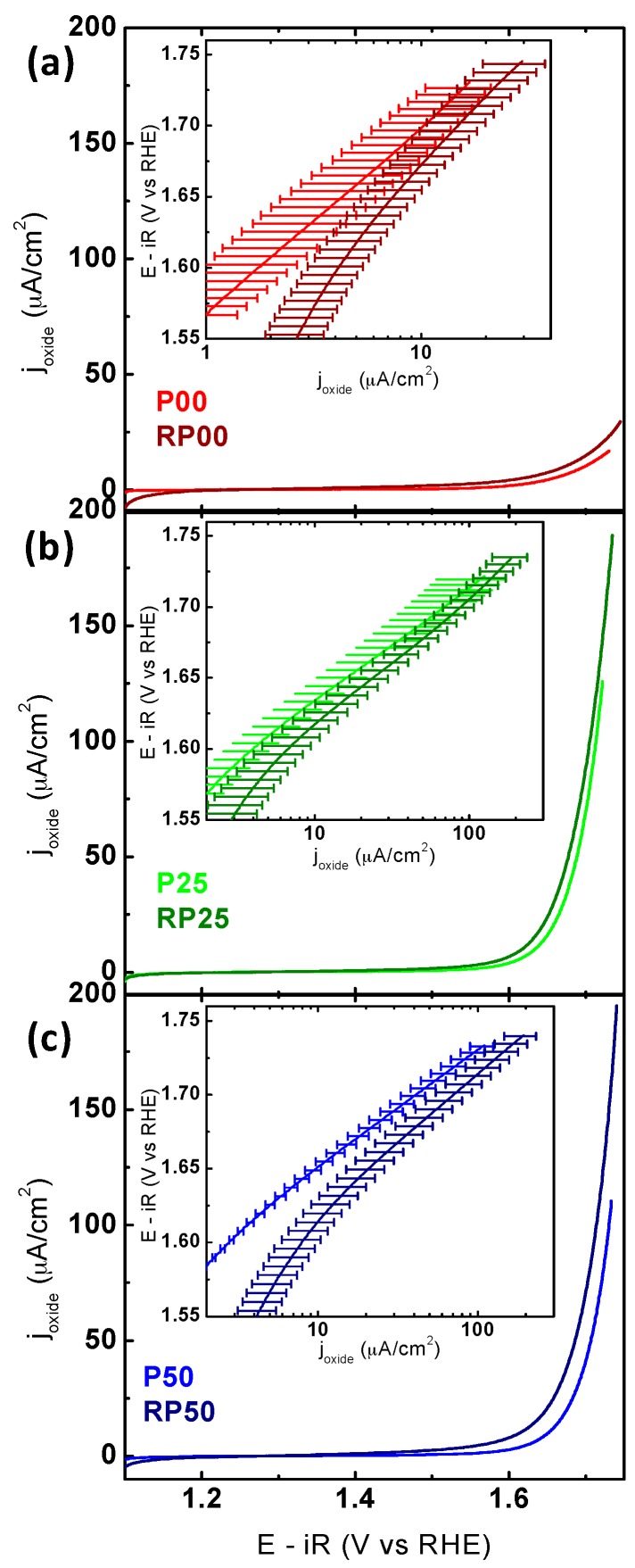
CV measurements comparing the oxygen evolution activity of P-PCMO and RP-PCMO for the same doping levels, respectively. (**a**) P00 and RP00; (**b**) P25 and RP25; (**c**) P50 and RP50. The insets show the Tafel analysis obtained from CV. The electrodes were prepared using an ink containing AB carbon and oxide particles with a loading of 0.4 mg_oxide_/cm^2^_disk_ on a glassy carbon electrode. All measurements were performed with O_2_-purged 0.1 M KOH supporting electrolyte at 10 mV/s and 1600 RPM. The voltage was corrected for electrolyte resistance and the averaged 5th scan is shown. At least two electrodes were averaged and the error bars indicate the standard deviation (for further information see Figure S2).

The OER activities of PCMO composite electrodes (P and RP-systems) were characterized using a rotating disk electrode in O_2_-saturated 0.1 M KOH solution. CV measurements of the 5th cycle averaged over at least two electrodes with the same respective stoichiometry are shown in Figure 5a–c (note the identical current density scale). The 5th cycle provides information about both the activity and stability as it best approximates the final activity (see discussion below). The apparent differences in the maximum applied potential are due to significant differences in the resistivity extracted by impedance spectroscopy and the subsequent potential correction.

Comparing the CVs obtained for the P- and RP-series demonstrates that the current densities of RP phase are consistently higher than that of the respective P-phase. The trend observed in the OER activity of samples is summarized in Table 3. The current densities at 1.65 V vs. RHE increased in the order of RP25~RP50~P25 > P50~RP00 > P00; however, error bars overlapped. The potential required to reach 50 μA·cm^−2^ obtained for two most active P and RP oxides was 1.69 V for both RP50 and P25. This overpotential is similar to that of La_0.5_Ca_0.5_MnO_3_ [48]. A comparison with the activity of other composite electrodes based on perovskite(-like) oxides is given in Table S2. Additionally, the currents in the CV were used to produce Tafel plots (the insets in Figure 5). The average Tafel slopes are lower for all P samples compared to the corresponding RP samples with identical doping level (see Figure S2 for determination of Tafel slopes). The average Tafel slopes of the Pr-free oxides were significantly larger than for the Pr-containing compounds (Table 3). Tafel slopes did not trend with electronic structure (Figure S2) and presumably have other origins (e.g., oxygen non-stoichiometry, multiple surface facets, carbon oxidation, etc.).

**Figure 6:**
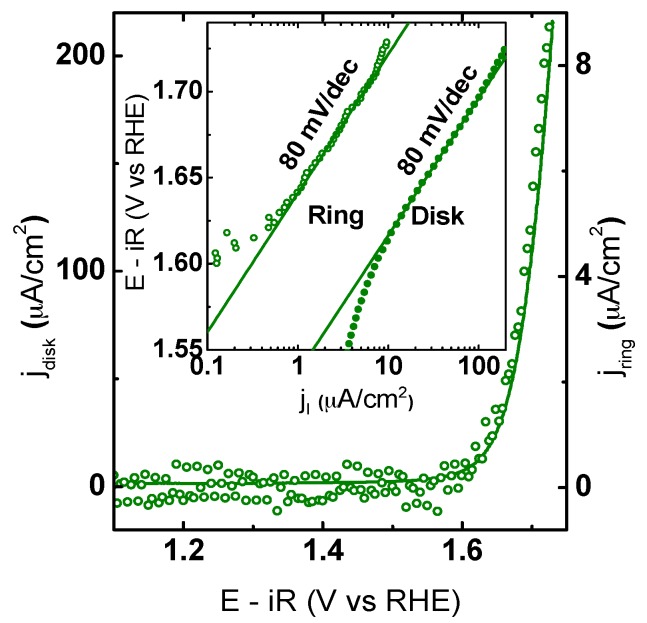
CV measurement of a composite electrode containing RP25, AB carbon (**solid** line) and the corresponding ring current (**open** circles) obtained by CA at 0.4 V vs. RHE. The inset shows the Tafel analysis of the Pt ring (**open** circles) and the disk RP25 (**solid** line) currents as a function of disk voltage obtained from CV. All measurements were performed with Ar-purged 0.1 M KOH supporting electrolyte at 10 mV/s and 1600 RPM. The voltage was corrected for electrolyte resistance and the positive-going direction of the 5th scan is shown. For the other samples, see Figure S3.

Additional RRDE experiments were performed in Ar-saturated electrolyte to verify OER activity. The Pt ring electrode was constantly held at 0.4 V vs. RHE to probe for O_2_. Ring CV measurements and calibration data are shown in Figure S3. In the RRDE experiments, oxygen generated at the disk moved to the ring by forced convection, where it was reduced under mass limiting conditions, i.e., the ring current only depends on concentration of oxygen. Under the used conditions, the ring currents solely responded to the dissolved oxygen [49]. Therefore, they represent the catalytic current and demonstrate that the exponentially rising currents on the disk of all electrodes above 1.6 V vs. RHE are indeed due to OER as can be seen in Figure 6.

Moreover, similar Tafel slopes of ring and disk current suggested that side reactions did not affect the disk currents of RP25, RP50, and P50 in this range (Figure S4). Significant deviations between the Tafel slope of ring and disk electrode indicates additional processes such as corrosion or possibly oxygen evolution from the oxide itself as Tafel slopes at the ring electrode are smaller than the respective disk Tafel slopes particularly for P00 (Figure S4).

**Figure 7:**
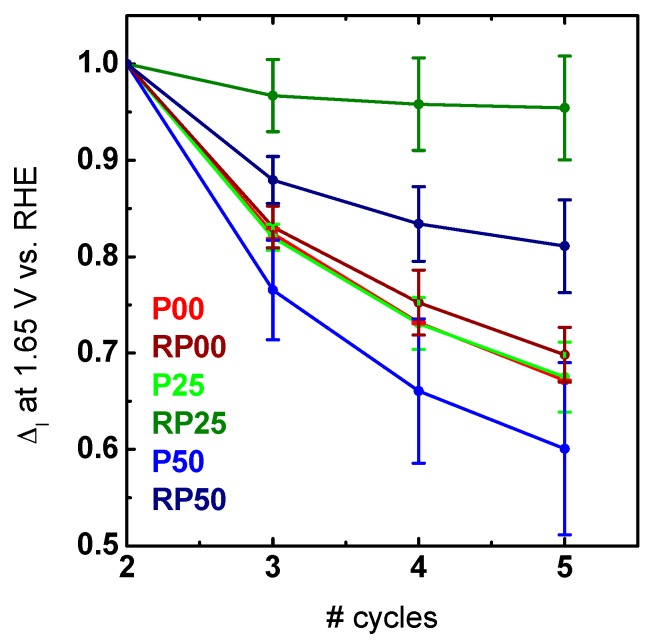
Relative changes in current density compared to the activity of the 2nd scan at a potential of 1.65 V vs. RHE of the averaged cyclic voltammograms. The 1st cycle was considered as condition treatment and therefore neglected for the relative changes.

In order to evaluate the stability of the different oxides, the change in the cyclic voltammograms was analyzed (Figure S5). Here we used the current density at a potential of 1.65 V vs. RHE relative to the second cycle shown in Figure 7. The potential was chosen to avoid the background currents such as the capacitance at low potentials on one hand and bubble formation at high potentials on the other hand. Pr-containing samples of the RP phase decayed less than the respective Pr-containing samples of the P-series (Figure 7). The difference in the stability even for the most active P sample might be due to additional processes as can be seen by the different Tafel slopes. Interestingly, the most active sample in our study (RP25, Table 3) was also the most stable one with a decrease of activity below 5%. The result of the most active in addition to be the most stable sample has been rarely observed and is in contrast to other reports [50,51] in which higher activity always results in lower stability.

### 2.4. Trends of OER Activity with Valence State and Covalency

**Figure 8:**
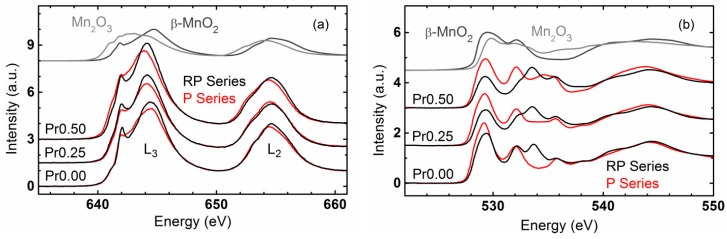
Total electron yield XANES of (**a**) Mn-L_3,2_ edges and (**b**) O-K edge of CaMnO_3_ (P00), Ca_0.75_Pr_0.25_MnO_3_ (P25), Ca_0.5_Pr_0.5_MnO_3_, (P50), Ca_2_MnO_4_ (RP00), Ca_1.75_Pr_0.25_MnO_4_ (RP25) and Ca_1.5_Pr_0.5_MnO_4_ (RP50). For reference, the spectra of Mn^III^_2_O_3_ and β-Mn^IV^O_2_ are also shown (with amplitude reduced by a factor of 3 for the Mn-L edges).

The surface electronic state of perovskites is assumed as a descriptor of the OER, where specifically the *e_g_* orbital filling of the TM cation and the covalency of the TM-O bond [20,48] are used in a rigid molecular orbital model. Herein, this method has been used for the study of a correlation between OER activity and orbital characteristics obtained by XAS experiments from our doping series of P- and RP-phases. Mn-L_3,2_ edge spectroscopy probes the manganese 2*p* to 3*d* electronic transitions. The Mn-L_3,2_ edge spectra of the P- and RP-systems exhibit shapes typical for those of mixed Mn^III/IV^ oxides, while differences only in minor details are observed in the samples with the same Pr-stoichiometry (Figure 8a). The Mn-L_3_ edges showed peaks at ~642 eV and ~645 eV as can be found in mixed Mn^III,IV^ oxides. Position, shape and height of these peaks depend on the ligand field-splitting parameter [52] and the fraction of Mn^III^ in the sample. The energy position of these peaks is insufficiently described by linear combination of the spectra of Mn^III^_2_O_3_ and β-Mn^IV^O_2_, which prompted us to perform further investigations on the electronic structure.

O-K edge spectroscopy probes the oxygen 1*s* to 2*p* electronic transition where the pre-edges are due to hybridization between transition metal 3*d* and oxygen 2*p* states. In contrast to the Mn-L edge spectra, the O-K pre-edges significantly differed for the samples with identical Pr-stoichiometry in the P- and RP-series (Figure 8b). Perovskite oxides (P-series) exhibited two peaks at ~529 eV and ~532 eV, while in the RP-series, a new peak at ~533.5 eV appeared. In addition, the latter peak faded into a shoulder with increasing Pr content. These differences observed in O-K edge indicated that the orbital degeneracy was further removed and consequently the orbitals in the RP-series split relative to the P-series. This is consistent with symmetry lowering of the MnO_6_ octahedra from a three-dimensional corner sharing a network in the P-systems with a two-dimensional network in the RP-systems with apical oxygen, pointing towards the AO_x_ layer with rock-salt structure, as discussed above. The apical oxygen site is singly coordinated to the Mn and exhibits a different (tetrahedral) A-site environment. For both P and RP-systems, the peak at lower energy (≈529 eV) can be assigned to *e_g_*^↑^ and *t*_2*g*_^↓^ states [53]. The lower conduction band edge is dominated by *e_g_*^↑^ and smoothly evolves into *t*_2*g*_^↓^ states with increasing energy without a minimum in the density of states. Therefore, both states cannot be separated experimentally. The assignment of the pre-edge features is shown in Figure S6. We will focus on the analysis of this peak in the following discussion.

Parameters of the electronic structure relating to *e_g_* orbitals depend on the crystal structure and the Pr stoichiometry. The Mn valence is determined based on calibration of the maximum in the Mn-L_3_ spectra to reference samples as shown in Figure S7. For the covalency factor, we used a method accessing only the hybridization of the O-2*p* and Mn-3*d* states which form the lower edge of the conduction band and thus represent the relevant acceptor states for OER. For the calculation of the covalency factor, the area of the *e_g_*^↑^/*t*_2*g*_^↓^ pre-peak at 529 eV in the O-K spectra was normalized by the total number of holes given by the experimentally derived Mn valence (Figure S8). This normalization accounts for the chemical effects of the oxygen stoichiometry. We note that our method differs from that published in reference [20], where additional peaks in the O-K pre-edge between 525 and 535 eV are included in the analysis (Figure S8) The first pre-peak area thus reflects density of states originating from the unoccupied O-2*p*–Mn-*d*_r2−z2_ σ-antibonds, the O-2*p*–Mn-*d*_x2−y2_ δ-bonds as well as hybridization of O-2*p* with Mn-d *t*_2*g*_^↓^ states. Spectral weight coming from Pr 4*f*, Mn-d *e_g_*^↓^ at higher energy and Ca d states is thus excluded.

**Figure 9:**
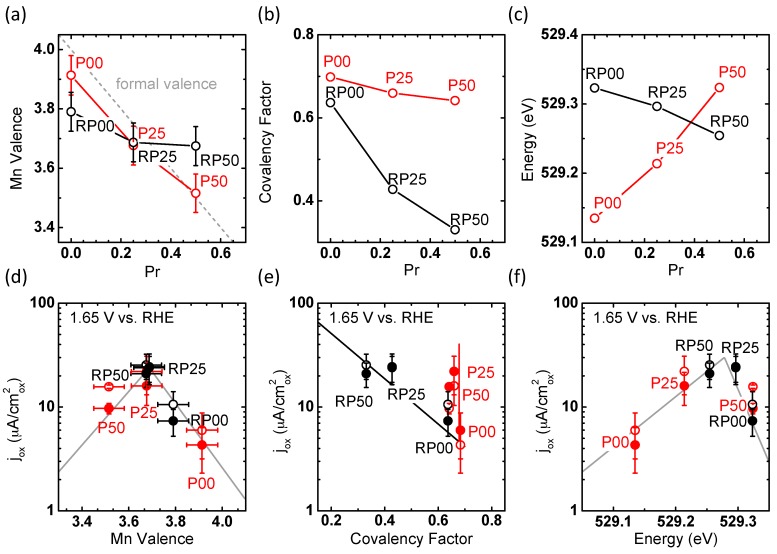
Effect of the Pr stoichiometry on (**a**) Mn valence determined from energy position of the maximum in the Mn-L_3_ spectra and formal valence from formula (dashed line); (**b**) covalency factor obtained as described in the text from the area of the *e_g_*^↑^/*t*_2*g*_^↓^ pre-peak in the O-K spectra; and (**c**) the energy position of the *e_g_*^↑^/*t*_2*g*_^↓^ pre-peak in the O-K spectra. Trend of OER current densities at 1.65 V vs. RHE with (**d**) Mn valence; (**e**) covalency factor; (**f**) energy position of the *e_g_*^↑^/*t*_2*g*_^↓^ pre-edge peak in the O-K spectra obtained during the 2nd (open circles) and 5th CV cycle (solid circles). Lines are guides to the eye.

The Mn valence of the P-samples was found within 0.1 units of the formal valence calculated from the chemical formula (Figure 9a). In contrast, the RP series exhibits a significantly reduced valence state for RP00. The Mn valence in the RP-system shows only little decrease with the increase of Pr content. This is in contrast to the covalency factor: It considerably decreases with the increase of Pr stoichiometry in the RP series, but only shows a slight decrease in the perovskite P-series (Figure 9b). Opposite trends are observed for the energy position of the *e_g_*^↑^/*t*_2*g*_^↓^ pre-peak in the O-K spectra of the P- and RP-series, respectively (Figure 9c). For perovskite samples (P-series), the energy increases from 529.13 to 529.32 eV with the increase of Pr stoichiometry, as expected for an increase of the Fermi level in a rigid band. In contrast, a decrease from 529.33 to 529.25 eV is observed for the RP-series. Therefore, the Fermi level shifts down despite of increasing nominal electron filling of the 3*d* band.

According to molecular orbital theory, the energy position of the antibonding states is shifted down by reducing the orbital overlap. Since the covalency factor is partially reflecting the orbital overlap of the Mn-3*d* *e_g_* and O-2*p* states, this is nicely confirmed by comparing Figure 9b,c. A pronounced change in covalency gives rise to a change in the character of the unoccupied Mn-3*d* *e_g_*–O-2*p* states and thus influences the charge state of the Mn ions. Thus, the strong change in covalency may partially explain the small changes of Mn valence state with Pr doping in the RP-series. In addition, the valence state of the RP00 particles is strongly influenced by oxygen vacancies, which is supported by the structural refinement presented above. In addition, Pr-rich stacking faults observed in PR50 via HR STEM (Figure 4e) can contribute to the excess valence of the remaining Pr-depleted RP layers. Their concentration can approach up to 20%. Furthermore, we cannot rule out that the determination of formal Mn valence state from Mn-L spectra is affected by the lift of the *e_g_* degeneracy in RP-PCMO.

Next, we discuss the observed correlation between the determined effective electronic parameters and the OER current density at 1.65 V (Figure 9d–f) during the 2nd and 5th cycles which allows discussion of stability in addition to OER activity. Trends in activity are often correlated with *e_g_* occupancy in recent literature. The total *e_g_* occupancy is calculated straightforwardly from the Mn valence, which is 1 for Mn^III^ and 0 for Mn^IV^ (i.e., *e_g_* = 4 − valence). Our samples show a maximum activity near a Mn valence of 3.7, i.e., a total *e_g_* occupancy of 0.3 (Figure 9d), while the optimum activity in a previous work on perovskite oxides [48] was obtained at *e_g_* filling of ≈1.2 (for a Co-Fe perovskite). Yet, the potential at 50 µA/cm^2^ of 1.68 ± 0.01 V vs. RHE for the RP25 system (0.3 *e_g_* electrons) is similar to 1.65 ± 0.03 V for La_0.5_Ca_0.5_MnO_3_ (nominally 0.5 *e_g_* electrons) in Suntivich et al. [48]. Thus, our work provides further evidence that the total *e_g_* occupancy is not solely a suitable activity descriptor [20,54]. Figure 9e suggests that in addition to Mn valence, the effect of varying the covalency factor must be taken into account. Here, it can only be analyzed for RP oxides having a similar valence state of 3.7 and thus similar total *e_g_* orbital occupancy of 0.3. For these samples, a lower covalency factor increases the OER activity (Figure 9e), which is inconsistent with the previous results for perovskite oxides at *e_g_* occupancy of ~1 [20,48].

This brings us to the discussion of an important difference of the orbital structure of the RP- compared to the P-systems studied in reference [48]. In both types of manganites, there is a doping-dependent splitting of the *e_g_* states, due to the Jahn-Teller distortion of Mn^3+^ species. This lifts the degeneracy of the two *e_g_*-type *d*_z2−r2_ and *d*_x2−y2_ orbitals as well as of the three *t_2g_*-type *d*_xy_, *d*_xz_ and *d*_yz_ orbitals. Since the Jahn-Teller distortion is absent for Mn^4+^, this type of splitting is not present in P00 and RP00 samples. XAS of the O-K edge can only access the upper unoccupied state of the Jahn Teller splitted *e_g_* doublet. Indeed, the shift of the O-K pre-edge peak position for the P-series reflects the reduced Jahn-Teller splitting with increasing Mn valence, similar to the trend observed by optical spectroscopy [53]. In the RP-system, the additional lift of degeneracy of the *e_g_* and *t*_2*g*_ states due to the stretched apical O(1)-Mn bond can be observed by the splitting of the unoccupied spin minority *e_g_*^↓^ states at ~532.5 and 533.5 eV which is only weakly doping dependent (Figure S6). This type of splitting of the *e_g_* state into *d*_z2−r2_ and *d*_x2−y2_ is dominated by the layered crystal structure.

**Figure 10:**
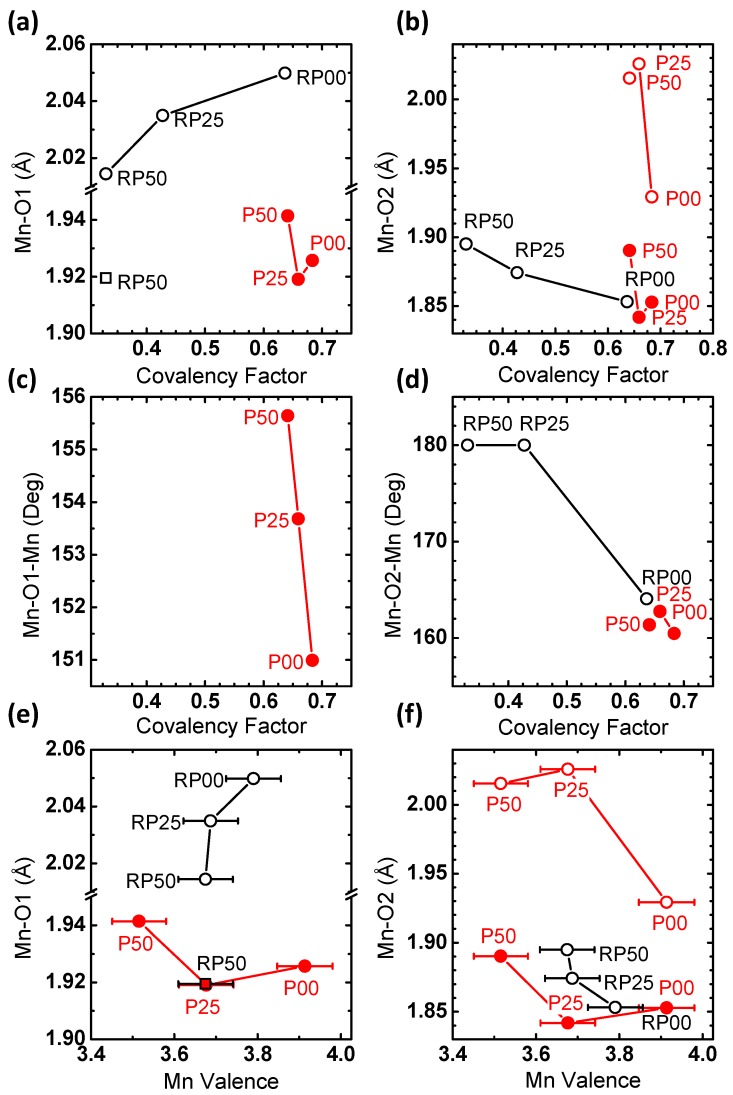
Correlation between Mn-O(1) and Mn-O(2) bond lengths, Mn-O(1)-Mn and Mn-O(2)-Mn bond angles as determined from Rietveld refinement with (**a**–**d**) covalency factor and (**e**,**f**) Mn valence.

In order to obtain a better understanding of the structure-property relations, Figure 10 depicts the correlation of the covalency factor and Mn valence with Mn-O bond distances and tilt angles. The covalency factor of the RP-series correlates with both the Mn-O(1) and Mn-O(2) distances, in contrast to the P-series (Figure 10a,b). For the RP-series, the covalency factor increases with the Mn-O(1) distance, because of the energy downshift of the unoccupied *d*_z2−r2_ which generally belongs to the largest bond length in the MnO_6_ octahedra. The Mn valence of the RP-series might also correlate with either bond distance, though valence changes were within error (Figure 10e,f). On the other hand, the Mn-O(1) and Mn-O(2) bond distances of the P-series agree with the degree of distortion expected for their valence [42] with the exception of P00, where pronounced octahedral distortions may be due to oxygen vacancies. The covalency factor increases with smaller angles of Mn-O(2)-Mn in Figure 10d, where a smaller angle corresponds to higher octahedral tilting. The trend of the longer Mn-O(2) bonds (open circles) follows that of the Mn-O(2)-Mn angle, while that of the shorter Mn-O(2) bonds (filled circles) is opposite (Figure 10b,d). Increasing octahedral tilting (i.e., lower angles) should reduce the orbital overlap between the O-2*p* and Mn-*d_r_*_2−z2_ σ-bonds and consequently should decrease the covalency. The result in Figure 10d is in contrast to such an expectation from σ-bonding. It may indicate a significant contribution of δ-type bonding of d_x2−y2_ orbitals which are oriented perpendicularly to the bonding axis to the O-K pre-edge feature. In addition, ab initio calculations in reference [53] show that the O-K pre-edge feature in P00 and P50 is affected by (minority spin) *t*_2*g*_^↓^ states in accordance with our assignment above.

Comparing the activity difference between the 2nd and 5th cycles in Figure 9e,f suggests that the stability is affected by the covalency rather than by the Mn valence. In fact, the samples bearing the smallest activity difference with cycling are found at a low covalency factor, while there are considerable stability differences across the investigated Mn valences. Figure 10d shows that the most stable samples of RP25 and RP50 are also characterized by a Mn-O(2)-Mn angle of 180°, i.e., untilted octahedra. Increasing octahedral tilting reduces the orbital overlap between the occupied O-2*p* and Mn-*d*_r2−z2_ σ-bonds and consequently weakens the bond strength. Simultaneously, reduced orbital overlap shifts down the unoccupied O-2*p*–Mn-*d*_r2−z2_ σ-antibonds, increases the spectral weights of O-2*p* and O-2*p*–Mn-*d*_x2−y2_ δ-contributions and thus strengthens the O-2*p* character of the acceptor states. Both might explain the reduced stability observed in samples with significant octahedral tilt. Note that due to their nature as occupied states, those suggested changes cannot be captured by O-K XAS.

Drawing from the above discussion of the individual influences of the valence and covalency on the activity as well as stability, we next introduce a combined observable. A clear volcano trend was observed between the OER activity and the energy position of the *e_g_*^↑^/*t*_2*g*_^↓^ pre-peak in the O-K spectra (Figure 9f). The trend obtained for both P- and RP-series yields both the highest current density (maximum activity) and stability at 529.25 eV for the sample RP25 at the top of the volcano, while other samples on either side of the volcano were less active and stable. As described above, the pre-peak onset energy is determined by two effects: Fermi level, i.e., doping (most clearly seen in the P-series) and the covalency, i.e., hybridization between unoccupied Mn-3*d* and O-2*p* states (most clearly seen in the RP-series). Based on the volcano-type relation between activity and pre-peak onset energy, we thus suggest that the energy position constitutes a suitable descriptor of the combined effect of valence and covalency. Such a suggestion has been supported by a statistical evaluation of different descriptors for OER [54], identifying valence and covalency as the two dominant influences on the OER activity.

Certainly, the suitability of combining both factors into one single quantity of the O-K onset energy needs more studies on other transition metal perovskites for verification. The physical picture following from such a descriptor is that a specific Mn valence has only the highest activity (Figure 9d) if contribution of oxygen states to the lower conduction band edge is close to a certain threshold value: Covalency should be large enough for facile electron transfer from adsorbed oxygen species to the MnO_6_ network; however, it should not be hampered by oxidation of lattice oxygen, which might initiate material degradation. Since covalency is not independent of band filling, a combined descriptor is suitable. Choosing O-K onset energy as the combined parameter is a first step for correlating the energy of a surface acceptor state for electron transfer to OER activity, in order to get a deeper insight into the electrochemical equilibrium of the Helmholtz layer and the required redox potentials for the electron transfer.

## 3. Materials and Methods

### 3.1. Synthesis Process

Pure phase samples of perovskite Ca_1−x_Pr_x_MnO_3_ (x = 0.00, 0.25, 0.50) have been prepared using a wet chemistry method. The precursors used for the synthesis of Ca_1−x_Pr_x_MnO_3_ nanoparticles were calcium nitrate tetrahydrate Ca(NO_3_)_2_·4H_2_O (99%), manganese nitrate tetrahydrate Mn(NO_3_)_2_·4H_2_O (99.5%), praseodymium nitrate hexahydrate Pr(NO_3_)_3_·6H_2_O (99.9%) and gelatin. Appropriate amounts of nitrates for 10 g of final product were dissolved in distilled water, stirring at room temperature for 20 min. Then, the 10 g gelatin solution stirred at 40 °C for 30 min was added to the solution of the cations and the whole solution was continually stirred at 60 °C for 2 h until it became clear with no precipitates or particulates. Then, a heat bath at 90 °C was used to evaporate the solvents until the desired resin-like product was obtained followed by drying at 200 °C in 5 min. The brownish black powder was calcined at 900 °C for 5 h.

Ruddelsden-Popper Ca_2−x_Pr_x_MnO_4_ (x = 0.00, 0.25, 0.50) samples were prepared by conventional solid-state reaction but with a novel approach starting from a stoichiometric mixture of Ca_1−x_Pr_x_MnO_3_ and CaO nanopowders. The reagents were first mixed in an agate mortar, ball-milled for 15 min and heated in air at 1100 °C for 24 h. In the following, the perovskites Ca_1__−__x_Pr_x_MnO_3_ at doping levels of x = 0.00, 0.25, 0.50 are abbreviated by P00, P25, P50 and likewise those of the layered (*n =* 1) RP-systems Ca_2−x_Pr_x_MnO_4_ are labeled RP00, RP25, RP50.

### 3.2. Characterizations

Powder X-ray diffraction (XRD) patterns were obtained with a Bruker D8 diffractometer (Karlsruhe, Germany) using monochromatized Cu Kα radiation. Phase identification and lattice parameters have been analyzed by Rietveld profile fitting using the program suite FullProf 2.00. The backgrounds in the Rietveld refinement were fitted with a 12-coefficient Fourier-cosine and the peak shapes were modeled by a pseudo-Voigt profile. Bond distances and angles were extracted using Bond_Str of the FullProf Suite (Version Jan 2015) and unit cell volumes were obtained using Powder Cell 2.4.

Scanning electron microscopy (SEM) was performed using an FEI Nova Nano SEM 650 (Hillsboro, OR, USA) operated at 5 and 15 keV, where a through-lens detector was used. The specific area of the particles was calculated by assuming an elliptical shape and defining an effective diameter as d_e_ = (d_2_ × d_1_)^0.5^, from which the specific area was obtained as A = 6/ρ Σd_e_^2^/Σd_e_^3^ [55]. The density ρ was calculated using the unit cell parameters in Table 1.

Transmission electron microscopy (TEM) analyses were obtained from the pellets prepared from synthesized powders. A piece of pellet was cut, mechanically polished using SiC paper and then finally thinned to electron transparency using a Gatan PIPS 691 ion polishing system (Munich, Germany). Transmission electron microscopy (TEM), electron diffraction (ED) and Scanning transmission electron (STEM) studies were performed using a FEI Titan 80-300 environmental microscope (Hillsboro, OR, USA) operated at 300 kV in high vacuum mode. The microscope is equipped with a Gatan Imaging Filter (GIF) Quantum 965ER used for electron energy-loss spectroscopy (EELS) experiments. Atomic models were built using the Vesta software package [56]. For the STEM imaging, high angle annular dark field contrast was used in order to visualize change in chemical composition.

Soft X-ray absorption spectroscopy (XAS) measurements at the Mn-L and O-K edges were performed at the spherical grating monochromator (SGM) beamline 11ID-1 at the Canadian Light Source [57]. Samples were prepared by covering carbon tape homogeneously with finely dispersed sample powders. The samples were mounted at an angle of roughly 45° with respect to both the incident beam and the detectors. All measurements were made at room temperature in the total electron yield mode. For the P- and RP-series samples, the incident energy was scanned continuously (slew scan mode). References were recorded in step scan mode during a previous beam time. All spectra were normalized by fitting a first-order polynomial in an appropriate region before the Mn-L_3_ edge or O-K pre-edges and subtracting it over the whole range of data. Subsequently, a second-order polynomial was fitted after the Mn-L_2_ edge or O-K edge and divided over the whole range of data to normalize the Mn-L_3_ Mn-L_2_ and O-K post-edges to unity. The energy axis was calibrated with respect to the pre-edge in the spectrum of molecular oxygen at 530.8 eV [58], which was acquired using a sample cell filled with ambient air.

The oxide electrodes for electrochemical measurements were prepared using a protocol published by Suntivich et al. [25] but omitting Nafion. The ink was prepared using acetylene black (AB) carbon (99.9+%, Alfa Aesar, Haverhill, MA, USA) treated in nitric acid overnight at 80 °C, subsequently filtered and dried at 100 °C. Additionally, tetrahydrofuran (THF, 99.9+%, Sigma Aldrich, Munich, Germany) and the respective oxide powder were added and sonicated for 30 min. The ink was composed of 1 mg/mL AB carbon and 5 mg/mL oxide particles. Finally, 2 × 5 µL of the ink were drop-cast on a freshly polished glassy carbon (GC) electrode (0.1257 cm^2^ area, ALS Co. Ltd., Tokyo, Japan) to yield 0.4 mg_ox_/cm^2^_disk_ oxide after loading carefully and controlling the drying to ensure a homogeneous coverage.

Electrochemical measurements were carried out with two Interface 1000E (Gamry Instruments Inc., Warminster, PA, USA) used as bipotentiostats assembled with a RRDE-3A rotator (ALS Co. Ltd.) in a glass cell using a three-electrode configuration at room temperature. The measurements were performed in 0.1 M KOH prepared from 1 M stock solution (Sigma Aldrich) and Milli-Q water (18.2 MΩ·cm) saturated with either O_2_ or Ar (99.999%, Air liquid, Kaufungen, Germany). The potentials were referenced to a saturated calomel electrode (ALS Co. Ltd.) calibrated to the reversible hydrogen electrode (RHE) scale by CV measurement of hydrogen evolution in H_2_-saturated 0.1 M KOH, where the average voltages of zero current from the positive and negative-going CV scans were found at 0.997 V vs. RHE. Additionally, the potentials were corrected for the electrolyte resistance extracted from the high frequency intercept of the real impedance measured by impedance spectroscopy at the disk. Cleanliness of the Pt ring was checked before each experiment by matching cyclic voltammograms (CV) with polycrystalline Pt [59]. CV measurements at the disk were carried out at 10 mV·s^−1^ and 1600 rpm. After CV measurements, chronoamperometry (CA) experiments were performed by increasing the potential from 1.70 to 1.75 V vs. RHE in steps of 0.05 V and keeping for 5 min. The ring voltage was always set to 0.4 V vs. RHE, where the current on ring is diffusion-limited. The currents measured on the prepared oxide electrode were corrected for the certain amount of the deposited oxide and corresponding surface area obtained using SEM analysis of the particle size distribution as described above.

## 4. Conclusions

The correlation between atomic structure, electronic properties, oxygen evolution activity and stability is presented for phase-pure nanoparticles of the perovskites Ca_1−x_Pr_x_MnO_3_ (P-PCMO) and the first-order Ruddlesden-Popper system Ca_2−x_Pr_x_MnO_4_ (RP-PCMO), with the A-site doping levels of x = 0.00, 0.25, 0.50, respectively. The crystal structure refined from X-ray powder diffraction shows the expected Pnma space group with strong octahedral tilting for P-PCMO. For the RP-PCMO, the space group changes with doping, possibly due to cation ordering and a change in oxygen vacancy concentration. Quite remarkably, the hybridization of unoccupied O-2*p* and Mn-3*d* states, described by the covalency factor, changes quite significantly with doping in RP-PCMO, while the changes in the formal Mn valence state are small. We attribute this to the lifting of the *e_g_* degeneracy due to the strong elongation of the apical Mn-O(1) bond. In contrast, for P-PCMO, the change in formal Mn valence state is close to the expected trend from doping and the covalency factor remains constant. The increase of covalency determined from the lower O-K pre-edge peak area of the *e_g_*^↑^/*t*_2*g*_^↓^ feature normalized to the number of Mn holes with increasing octahedral tilt angle is contrary to the expectation from Mn-*e_g_*–O-2*p* σ-type bonding and may reflect the contribution of δ-type bonding of the d_x2−y2_ orbitals which are oriented perpendicularly to the bonding axis, as well as the contribution of minority *t*_2*g*_^↓^ states to the O-K pre-edge feature to the conduction band. Careful electrochemical characterization by cyclic voltammetry shows improved activity and stability of RP-PCMO as compared to P-PCMO and Pr vs. non-doped systems. All but RP25 and RP50 degrade equally fast in Figure 7.

Remarkably, our data shows that both highest activity and stability is observed at a Mn valence state of 3.7 when the covalency factor has a relative minimum. This observation suggests that oxygen redox processes and related oxygen vacancy formation may be less pronounced for manganites with reduced covalency of the unoccupied states. This may be a hint that oxidation of lattice oxygen due to a too-strong O-2*p* character of the empty states may be unfavorable, at least for manganites, and suggests that keeping redox processes at the Mn sites is favorable. Since valence and covalency changes are not entirely independent, the combined effect of the Mn valence and O-Mn covalency change is expressed via the energy position of the *e_g_*^↑^/*t*_2*g*_^↓^ pre-edge peak in the O-K spectra as a suggested new descriptor, leading to a volcano-like representation of the oxygen evolution reaction (OER) activity.

## Figures and Tables

**Table 1:** Lattice parameters of the perovskite manganites P00, P25 and P50 as well as the RP phases RP00, RP25 and RP50 at different Pr-doping levels.

System	Composition	Space Group	a (Å)	b (Å)	c (Å)	V (Å^3^)	Density (g/cm^3^)
P00	CaMnO_3_	Pnma	5.275	7.457	5.267	207.190	4.585
P25	Ca_0.75_Pr_0.25_MnO_3_	Pnma	5.328	7.589	5.325	215.302	5.190
P50	Ca_0.50_Pr_0.50_MnO_3_	Pnma	5.404	7.663	5.395	223.392	5.751
RP00	Ca_2_MnO_4_	I 41/acd	5.191	5.191	24.082	648.858	4.074
RP25	Ca_1.75_Pr_0.25_MnO_4_	I 4/mmm	3.748	3.748	11.880	166.926	4.463
RP50	Ca_1.50_Pr_0.50_MnO_4_	Fmm2	5.365	5.354	11.840	340.095	4.872

**Table 2:** Mn-O bond distance and Mn-O-Mn bond angles for the perovskite manganites P00, P25 and P50 as well as the RP phases RP00, RP25 and RP50.

System	Composition	Mn-O(1)	Mn-O(2)	Mn-O(1)-Mn	Mn-O(2)-Mn
P00	CaMnO_3_	1.926	1.853, 1.929	151.0	160.5
P25	Ca_0.75_Pr_0.25_MnO_3_	1.919	1.842, 2.026	153.7	162.7
P50	Ca_0.50_Pr_0.50_MnO_3_	1.941	1.890, 2.015	155.6	161.4
RP00	Ca_2_MnO_4_	2.050	1.853	–	164.1
RP25	Ca_1.75_Pr_0.25_MnO_4_	2.035	1.874	–	180.0
RP50	Ca_1.50_Pr_0.50_MnO_4_	2.014, 1.920 *	1.895	–	180.0

* Mn-O(3) located on apical site.

**Table 3:** Electrochemical quantities determined from cyclic voltammetry (CV). The Tafel slopes were determined for each capacitance-corrected CV (5th cycle) and then averaged for all respective electrodes.

System	Tafel Slope (CV)	V vs. RHE (10 μA/cm^2^)	V vs. RHE (50 µA/cm^2^)	j_oxide_ (1.65 V vs. RHE) (μA/cm^2^)
P00	130 ± 7	1.70 ± 0.02	n/a	4.3 ± 2.0
P25	79 ± 4	1.63 ± 0.01	1.69 ± 0.02	16.0 ± 5.7
P50	80 ± 3	1.65 ± 0.01	1.71 ± 0.01	9.7 ± 1.2
RP00	165 ± 6	1.68 ± 0.01	n/a	7.4 ± 2.1
RP25	86 ± 8	1.62 ± 0.01	1.68 ± 0.01	24.0 ± 6.7
RP50	93 ± 4	1.61 ± 0.02	1.69 ± 0.01	21.0 ± 5.6

## Supplementary Materials

The following are available online at www.mdpi.com/1996-1944/9/11/921/s1: Figures S1–S8, Tables S1 and S2 and cif-files of P00, P25, P50, RP00, RP25, RP50.

## References

[1] Bockris J.O.M., Otagawa T. (1984). The electrocatalysis of oxygen evolution on perovskites. J. Electrochem. Soc..

[2] Bockris J.O., Otagawa T. (1983). Mechanism of oxygen evolution on perovskites. J. Phys. Chem..

[3] Matsumoto Y., Sato E. (1986). Electrocatalytic properties of transition metal oxides for oxygen evolution reaction. Mater. Chem. Phys..

[4] Bockris J.O.M. (1956). Kinetics of Activation Controlled Consecutive Electrochemical Reactions: Anodic Evolution of Oxygen. J. Chem. Phys..

[5] Hardin W.G., Mefford J.T., Slanac D.A., Patel B.B., Wang X., Dai S., Zhao X., Ruoff R.S., Johnston K.P., Stevenson K.J. (2014). Tuning the Electrocatalytic Activity of Perovskites through Active Site Variation and Support Interactions. Chem. Mater..

[6] Hardin W.G., Slanac D.A., Wang X., Dai S., Johnston K.P., Stevenson K.J. (2013). Highly Active, Nonprecious Metal Perovskite Electrocatalysts for Bifunctional Metal–Air Battery Electrodes. J. Phys. Chem. Lett..

[7] Vojvodic A., Nørskov J.K. (2011). Optimizing Perovskites for the Water-Splitting Reaction. Science.

[8] Seo M.H., Park H.W., Lee D.U., Park M.G., Chen Z. (2015). Design of Highly Active Perovskite Oxides for Oxygen Evolution Reaction by Combining Experimental and ab Initio Studies. ACS Catal..

[9] Trasatti S. (1980). Electrocatalysis by oxides—Attempt at a unifying approach. J. Electroanal. Chem. Interfacial Electrochem..

[10] Smith R.D.L., Prévot M.S., Fagan R.D., Zhang Z., Sedach P.A., Siu M.K.J., Trudel S., Berlinguette C.P. (2013). Photochemical Route for Accessing Amorphous Metal Oxide Materials for Water Oxidation Catalysis. Science.

[11] Zhang C., Trudel S., Berlinguette C.P. (2014). Water Oxidation Catalysis: Survey of Amorphous Binary Metal Oxide Films Containing Lanthanum and Late 3d Transition Metals. Eur. J. Inorgan. Chem..

[12] Zhang Z., Liu J., Gu J., Su L., Cheng L. (2014). An overview of metal oxide materials as electrocatalysts and supports for polymer electrolyte fuel cells. Energy Environ. Sci..

[13] Trotochaud L., Ranney J.K., Williams K.N., Boettcher S.W. (2012). Solution-Cast Metal Oxide Thin Film Electrocatalysts for Oxygen Evolution. J. Am. Chem. Soc..

[14] Doyle R.L., Godwin I.J., Brandon M.P., Lyons M.E.G. (2013). Redox and electrochemical water splitting catalytic properties of hydrated metal oxide modified electrodes. Phys. Chem. Chem. Phys..

[15] Hong W.T., Risch M., Stoerzinger K.A., Grimaud A., Suntivich J., Shao-Horn Y. (2015). Toward the rational design of non-precious transition metal oxides for oxygen electrocatalysis. Energy Environ. Sci..

[16] Mom R.V., Cheng J., Koper M.T.M., Sprik M. (2014). Modeling the Oxygen Evolution Reaction on Metal Oxides: The Infuence of Unrestricted DFT Calculations. J. Phys. Chem. C.

[17] Su H.-Y., Gorlin Y., Man I.C., Calle-Vallejo F., Norskov J.K., Jaramillo T.F., Rossmeisl J. (2012). Identifying active surface phases for metal oxide electrocatalysts: A study of manganese oxide bi-functional catalysts for oxygen reduction and water oxidation catalysis. Phys. Chem. Chem. Phys..

[18] Arrigo R., Hävecker M., Schuster M.E., Ranjan C., Stotz E., Knop-Gericke A., Schlögl R. (2013). In Situ Study of the Gas-Phase Electrolysis of Water on Platinum by NAP-XPS. Angew. Chem. Int. Ed..

[19] Torrance J.B., Lacorre P., Asavaroengchai C., Metzger R.M. (1991). Why are some oxides metallic, while most are insulating?. Phys. C Supercond..

[20] Suntivich J., Hong W.T., Lee Y.-L., Rondinelli J.M., Yang W., Goodenough J.B., Dabrowski B., Freeland J.W., Shao-Horn Y. (2014). Estimating Hybridization of Transition Metal and Oxygen States in Perovskites from O K-edge X-ray Absorption Spectroscopy. J. Phys. Chem. C.

[21] Alghamdi K., Hargreaves J.S.J., Jackson S.D. (2009). Base Catalysis with Metal Oxides. Metal Oxide Catalysis.

[22] Rossmeisl J., Qu Z.W., Zhu H., Kroes G.J., Nørskov J.K. (2007). Electrolysis of water on oxide surfaces. J. Electroanal. Chem..

[23] Rossmeisl J., Logadottir A., Nørskov J.K. (2005). Electrolysis of water on (oxidized) metal surfaces. Chem. Phys..

[24] Raabe S., Mierwaldt D., Ciston J., Uijttewaal M., Stein H., Hoffmann J., Zhu Y., Blöchl P., Jooss C. (2012). In Situ Electrochemical Electron Microscopy Study of Oxygen Evolution Activity of Doped Manganite Perovskites. Adv. Funct. Mater..

[25] Suntivich J., Gasteiger H.A., Yabuuchi N., Shao-Horn Y. (2010). Electrocatalytic Measurement Methodology of Oxide Catalysts Using a Thin-Film Rotating Disk Electrode. J. Electrochem. Soc..

[26] May K.J., Carlton C.E., Stoerzinger K.A., Risch M., Suntivich J., Lee Y.-L., Grimaud A., Shao-Horn Y. (2012). Influence of Oxygen Evolution during Water Oxidation on the Surface of Perovskite Oxide Catalysts. J. Phys. Chem. Lett..

[27] Risch M., Grimaud A., May K.J., Stoerzinger K.A., Chen T.J., Mansour A.N., Shao-Horn Y. (2013). Structural Changes of Cobalt-Based Perovskites upon Water Oxidation Investigated by EXAFS. J. Phys. Chem. C.

[28] Grimaud A., May K.J., Carlton C.E., Lee Y.-L., Risch M., Hong W.T., Zhou J., Shao-Horn Y. (2013). Double perovskites as a family of highly active catalysts for oxygen evolution in alkaline solution. Nat. Commun..

[29] Bassat J.-M., Burriel M., Wahyudi O., Castaing R., Ceretti M., Veber P., Weill I., Villesuzanne A., Grenier J.-C., Paulus W. (2013). Anisotropic Oxygen Diffusion Properties in Pr_2_NiO_4+δ_ and Nd_2_NiO_4+δ_ Single Crystals. J. Phys. Chem. C.

[30] Nakamura T., Yashiro K., Sato K., Mizusaki J. (2009). Oxygen nonstoichiometry and defect equilibrium in La_2−x_Sr_x_NiO_4+δ_. Solid State Ion..

[31] Vashook V.V., Tolochko S.P., Yushkevich I.I., Makhnach L.V., Kononyuk I.F., Altenburg H., Hauck J., Ullmann H. (1998). Oxygen nonstoichiometry and electrical conductivity of the solid solutions La_2−x_Sr_x_NiO_y_ (0 ≤ x ≤ 0.5). Solid State Ion..

[32] Nakamura T., Ling Y., Amezawa K. (2015). The effect of interstitial oxygen formation on the crystal lattice deformation in layered perovskite oxides for electrochemical devices. J. Mater. Chem. A.

[33] Grimaud A., Hong W.T., Shao-Horn Y., Tarascon J.M. (2016). Anionic redox processes for electrochemical devices. Nat. Mater..

[34] Rong X., Parolin J., Kolpak A.M. (2016). A Fundamental Relationship between Reaction Mechanism and Stability in Metal Oxide Catalysts for Oxygen Evolution. ACS Catal..

[35] Mefford J.T., Rong X., Abakumov A.M., Hardin W.G., Dai S., Kolpak A.M., Johnston K.P., Stevenson K.J. (2016). Water electrolysis on La_1−x_Sr_x_CoO_3−δ_ perovskite electrocatalysts. Nat. Commun..

[36] Mildner S., Beleggia M., Mierwaldt D., Hansen T.W., Wagner J.B., Yazdi S., Kasama T., Ciston J., Zhu Y., Jooss C. (2015). Environmental TEM Study of Electron Beam Induced Electrochemistry of Pr_0.64_Ca_0.36_MnO_3_ Catalysts for Oxygen Evolution. J. Phys. Chem. C.

[37] Mierwaldt D., Mildner S., Arrigo R., Knop-Gericke A., Franke E., Blumenstein A., Hoffmann J., Jooss C. (2014). In Situ XANES/XPS Investigation of Doped Manganese Perovskite Catalysts. Catalysts.

[38] Umena Y., Kawakami K., Shen J.-R., Kamiya N. (2011). Crystal structure of oxygen-evolving photosystem II at a resolution of 1.9 Å. Nature.

[39] Post J.E. (1999). Manganese oxide minerals: Crystal structures and economic and environmental significance. Proc. Natl. Acad. Sci. USA.

[40] Siegbahn P.E.M. (2012). Mechanisms for proton release during water oxidation in the S2 to S3 and S3 to S4 transitions in photosystem II. Phys. Chem. Chem. Phys..

[41] Fawcett I.D., Sunstrom J.E., Greenblatt M., Croft M., Ramanujachary K.V. (1998). Structure, Magnetism, and Properties of Ruddlesden−Popper Calcium Manganates Prepared from Citrate Gels. Chem. Mater..

[42] Jirák Z., Krupička S., Šimša Z., Dlouhá M., Vratislav S. (1985). Neutron diffraction study of Pr_1−x_Ca_x_MnO_3_ perovskites. J. Magn. Magn. Mater..

[43] Shannon R. (1976). Revised effective ionic radii and systematic studies of interatomic distances in halides and chalcogenides. Acta Crystallogr. Sect. A.

[44] Daoudi A., Le Flem G. (1972). Sur une série de solutions solides de formule Ca_2−x_Ln_x_MnO_4_ (L*n =* Pr, Nd, Sm, Eu, Gd). J. Solid State Chem..

[45] Ibarra M., Retoux R., Hervieu M., Autret C., Maignan A., Martin C., Raveau B. (2003). Charge–orbital ordering above room temperature in the 2D Pr_1−x_Ca_1+x_MnO_4_ manganites. J. Solid State Chem..

[46] Chen D., Chen C., Baiyee Z.M., Shao Z., Ciucci F. (2015). Nonstoichiometric oxides as low-cost and highly-efficient oxygen reduction/evolution catalysts for low-temperature electrochemical devices. Chem. Rev..

[47] Aschauer U., Pfenninger R., Selbach S.M., Grande T., Spaldin N.A. (2013). Strain-controlled oxygen vacancy formation and ordering in CaMnO_3_. Phys. Rev. B.

[48] Suntivich J., May K.J., Gasteiger H.A., Goodenough J.B., Shao-Horn Y. (2011). A Perovskite Oxide Optimized for Oxygen Evolution Catalysis from Molecular Orbital Principles. Science.

[49] Scholz J., Risch M., Stoerzinger K.A., Wartner G., Jooss Ch. (2016). Rotating Ring Disk Study of Oxygen Evolution at a Perovskite Surface: Correlating Activity to Manganese Concentration. J. Phys. Chem. C.

[50] Binninger T., Mohamed R., Waltar K., Fabbri E., Levecque P., Kötz R., Schmidt T.J. (2015). Thermodynamic explanation of the universal correlation between oxygen evolution activity and corrosion of oxide catalysts. Sci. Rep..

[51] Danilovic N., Subbaraman R., Chang K.-C., Chang S.H., Kang Y.J., Snyder J., Paulikas A.P., Strmcnik D., Kim Y.-T., Myers D. (2014). Activity–Stability Trends for the Oxygen Evolution Reaction on Monometallic Oxides in Acidic Environments. J. Phys. Chem. Lett..

[52] De Groot F.M.F., Fuggle J.C., Thole B.T., Sawatzky G.A. (1990). 2*p* X-ray absorption of 3d transition-metal compounds: An atomic multiplet description including the crystal field. Phys. Rev. B.

[53] Mildner S., Hoffmann J., Blöchl P.E., Techert S., Jooss C. (2015). Temperature- and doping-dependent optical absorption in the small-polaron system Pr_1−x_Ca_x_MnO_3_. Phys. Rev. B.

[54] Hong W.T., Welsch R.E., Shao-Horn Y. (2016). Descriptors of Oxygen-Evolution Activity for Oxides: A Statistical Evaluation. J. Phys. Chem. C.

[55] Trasatti S., Petrii O.A. (1992). Real surface area measurements in electrochemistry. J. Electroanal. Chem..

[56] Momma K., Izumi F. (2008). VESTA: A three-dimensional visualization system for electronic and structural analysis. J. Appl. Crystallogr..

[57] Regier T., Krochak J., Sham T.K., Hu Y.F., Thompson J., Blyth R.I.R. (2007). Performance and capabilities of the Canadian Dragon: The SGM beamline at the Canadian Light Source. Nucl. Instrum. Methods Phys. Res. Sect. A Accel. Spectrom. Detect. Assoc. Equip..

[58] Hitchcock A.P., Brion C.E. (1980). K-shell excitation spectra of CO, N_2_ and O_2_. J. Electron Spectrosc. Relat. Phenom..

[59] Rheinländer P., Henning S., Herranz J., Gasteiger H.A. (2013). Comparing hydrogen oxidation and evolution reaction kinetics on polycrystalline platinum in 0.1 M and 1 M KOH. ECS Trans..

