# Supplementary material for: Oxygen Evolution at Manganite Perovskite Ruddlesden-Popper Type Particles: Trends of Activity on Structure, Valence and Covalence
Source: Materials (Basel). 2016 Nov 14;9(11):921. doi: 10.3390/ma9110921 (PMC5457185; doi:10.3390/ma9110921)
Supplement: Supplementary file 1 [file materials-09-00921-s001.pdf]

Supplementary Materials: Oxygen Evolution at Manganite Perovskite Ruddlesden-Popper Type Particles: Trends of Activity on Structure, Valence and Covalence

Majid Ebrahimizadeh Abrishami, Marcel Risch, Julius Scholz, Vladimir Roddatis, Norbert Osterthun and Christian Jooss

A. Particle Size Distributions

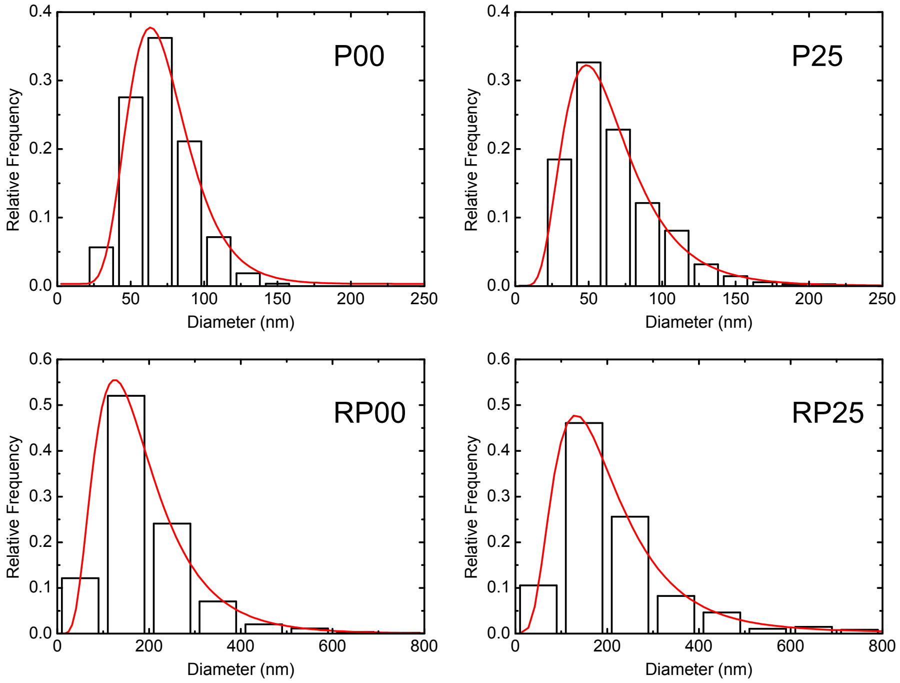

Figure S1. Histograms for particle size distribution calculated by assuming the elliptical shape of the particles and calculating the effective diameter as $d_e = (d_2 \times d_1)^{0.5}$. Size distributions for the P50 and RP50 systems are shown in the main text.

Table S1. Diameters and specific area obtained by SEM analysis. Histograms can be found in Figure 3c,d and Figure S1.

Sample	Effective Diameter (nm)	Density (g/cm³)	Specific Area (cm²/mg)
P00	70 ± 22	4.585	81.63
P25	65 ± 30	5.190	63.45
P50	64 ± 33	5.751	49.96
RP00	198 ± 133	4.074	16.12
RP25	223 ± 153	4.463	18.04
RP50	398 ± 200	4.872	12.01

B. Electrochemical Characterization via Rotating Ring Disc Electrode

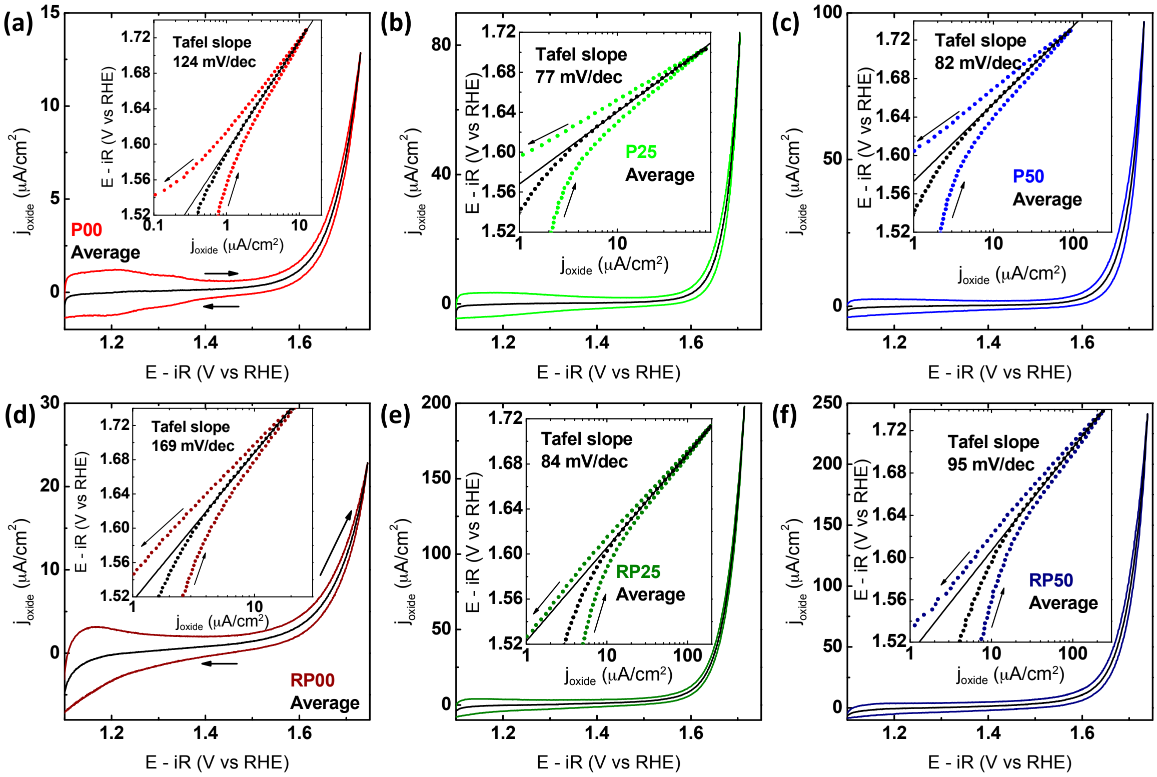

Figure S2. Representative CV measurements showing the raw disk data and, in black, averaged currents from both the forward and backward scan direction indicated by the arrows. (**a**–**c**) show the oxygen evolution activity of the P- and (**d**–**f**) of the RP-series. The insets show the Tafel analysis obtained from CV. The electrodes were prepared using an ink containing AB carbon and oxide particles with a loading of 0.4 mg_{oxide}/cm^2_{disk} on a glassy carbon electrode. All measurements were performed with O_2-purged 0.1 M KOH supporting electrolyte at 10 mV/s and 1600 RPM. The voltage was corrected for electrolyte resistance and the 5th scan is shown. The currents of forward and backward scan direction were averaged for all electrodes of the same respective stoichiometry.

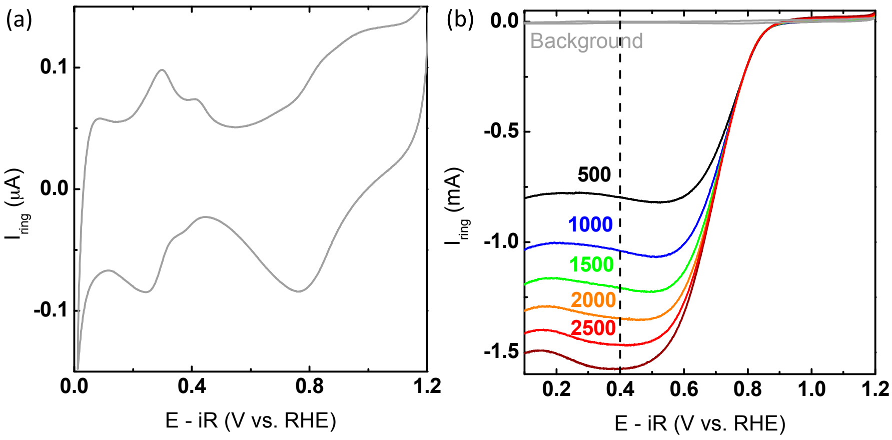

Figure S3. (**a**) Unrotated CV measurement of the freshly polished Pt-ring in Ar-saturated 0.1 M KOH supporting electrolyte at 200 mV/s showing the expected features of a polycrystalline Pt metal without surface oxides [1]; (**b**) Ring currents of a freshly polished Pt-ring in 0.1 M KOH supporting electrolyte (Ar-purged for the background measurement, O_2-purged for ORR measurements) at 200 mV/s. Rotation speeds were increased from 500 to 3000 rpm (lines). All measurements shown are the forward direction of the 2nd cycle.

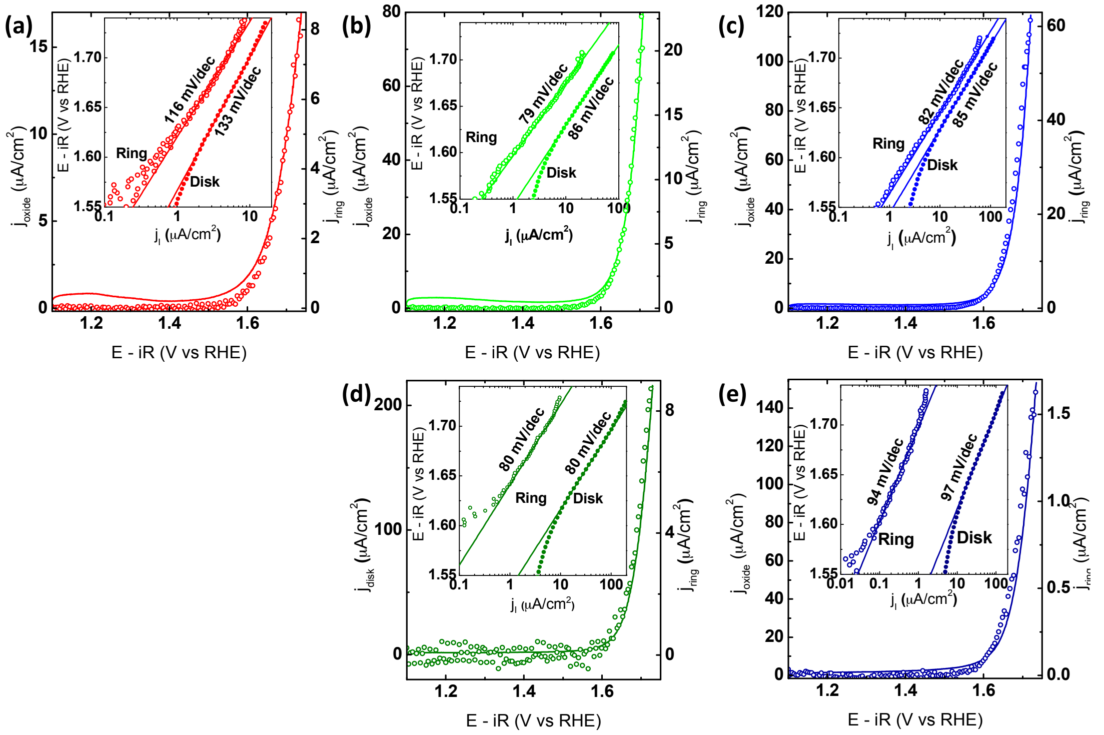

Figure S4. RRDE CV measurements of all composite electrodes (**a**) P00; (**b**) P25; (**c**) P50; (**d**) RP25; (**e**) RP50 (solid lines) and their corresponding ring currents (open circles) obtained by CA at 0.4 V vs. RHE. The insets show the Tafel analysis of the Pt ring (open circles) and the respective disk (solid line) currents as a function of disk voltage obtained from CV. All measurements were performed with Ar-purged 0.1 M KOH supporting electrolyte at 10 mV/s and 1600 RPM. The voltage was corrected for electrolyte resistance and the positive-going direction of the 5th scan is shown. For the RP00 system, the available data sets did not allow for detailed analysis.

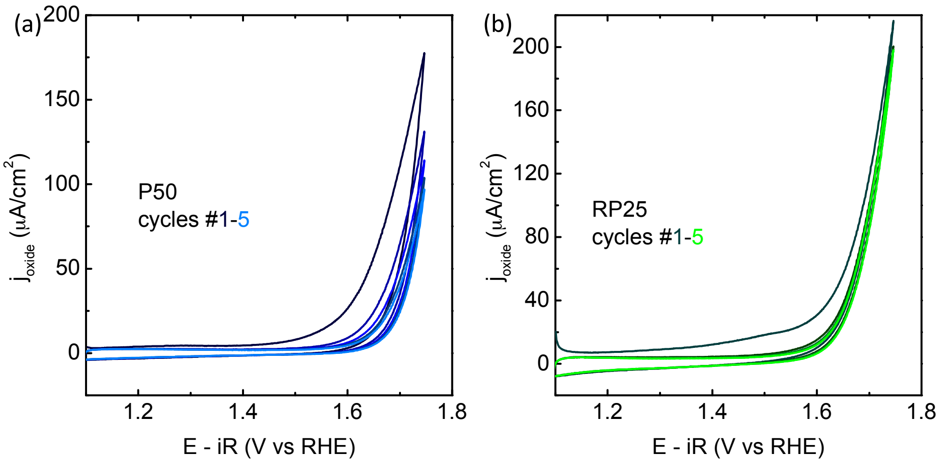

Figure S5. Representative CV measurements showing the first five cycles in (**a**) for P50 (the system with the most pronounced decrease in activity) and (**b**) for RP25 (the system with the most stable activity). The electrodes were prepared using an ink containing AB carbon and oxide particles with a loading of 0.4 mg_{oxide}/cm^2_{disk} on a glassy carbon electrode. All measurements were performed with O_2-purged 0.1 M KOH supporting electrolyte at 10 mV/s and 1600 RPM. The voltage was corrected for electrolyte resistance.

Table S2. OER overpotentials at 50 uA/cm$^2_{oxide}$.

Sample	Overpotential (V)	E vs. RHE (V)	e_g	Reference
$LaMnO_3$	0.40 ± 0.01	1.63 ± 0.01	1.0	[2]
$LaMnO_{3+\delta}$	0.36 ± 0.03	1.59 ± 0.03	0.8	[2]
$La_{0.5}Ca_{0.5}MnO_3$	0.42 ± 0.03	1.65 ± 0.03	0.5	[2]
$LaMn_{0.5}Cu_{0.5}O_3$	0.39 ± 0.02	1.62 ± 0.02	0.3	[2]
$LaCoO_3$	0.35 ± 0.02	1.58 ± 0.02	1.0	[2]
$La_{0.5}Ca_{0.5}CoO_3$	0.29 ± 0.02	1.52 ± 0.02	1.5	[2]
$Ba_{0.5}Sr_{0.5}Co_{0.8}Fe_{0.2}O_{3-\delta}$	0.25 ± 0.02	1.48 ± 0.02	1.3	[2]
$La_{0.8}Sr_{0.2}MnO_3$	0.44	1.67	0.8	[3]
$La_{0.4}Sr_{0.6}CoO_3$	0.37	1.60	1.4	[4]
P25	0.46 ± 0.02	1.69 ± 0.02	0.3	This work
P50	0.48 ± 0.01	1.71 ± 0.01	0.5	This work
RP25	0.45 ± 0.01	1.68 ± 0.01	0.3	This work
RP50	0.46 ± 0.01	1.69 ± 0.01	0.3	This work

C. X-ray Absorption Spectroscopy

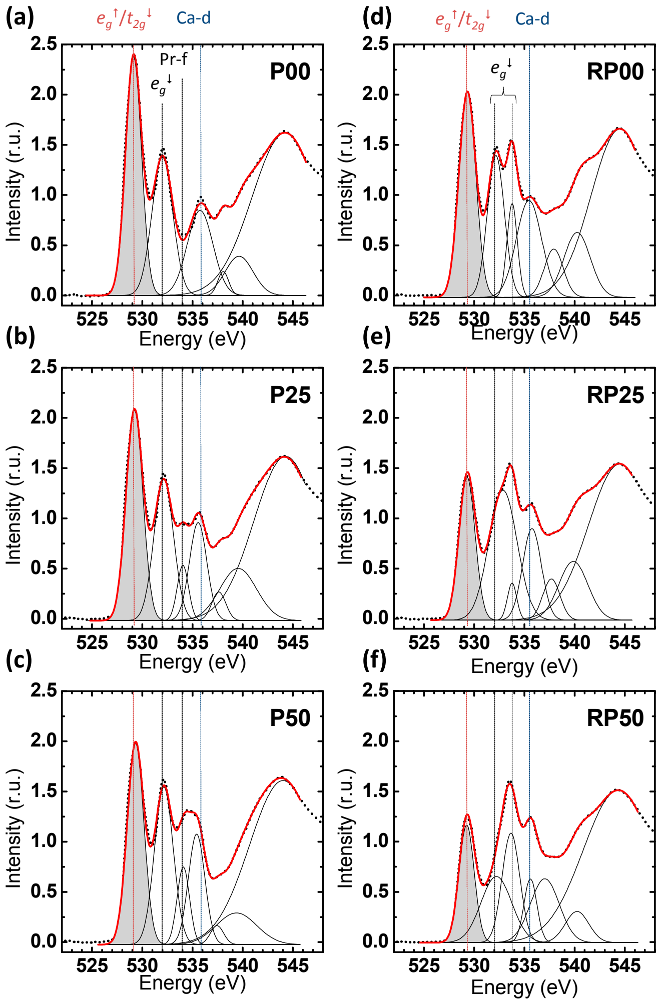

Figure S6. Fits of the O-K pre-edge using Gaussians (black) and their sum (red) for the P- (**a**–**c**) and the RP-series (**d**–**f**), respectively. Fitting was performed in Origin 8.5 using the function "fitpeaks". Assignments of the peaks for the P-series have been done according to the ab initio calculations in reference [5]. The area and position of the shaded peak is correlated with OER activity in the main text.

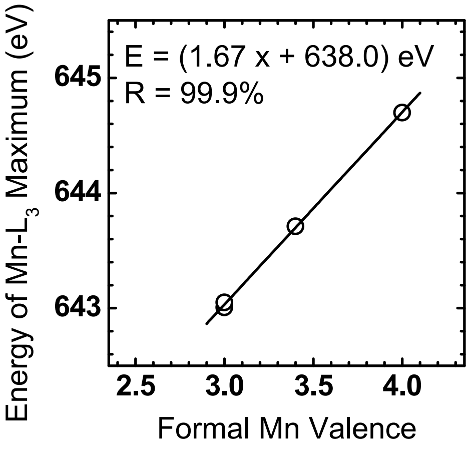

Figure S7. Valence state calibration using the maxima of the Mn-L3 edges of references with known Mn valence: $PrMn^{3+}O_3$, $Mn^{3+}{}_2O_3$, $La_{0.6}Sr_{0.4}Mn^{3.4+}O_3$ and β-$Mn^{4+}O_2$ (black circles). We note that the valence calibration via the energy position of the peak maximum becomes inaccurate below Mn^{3+}.

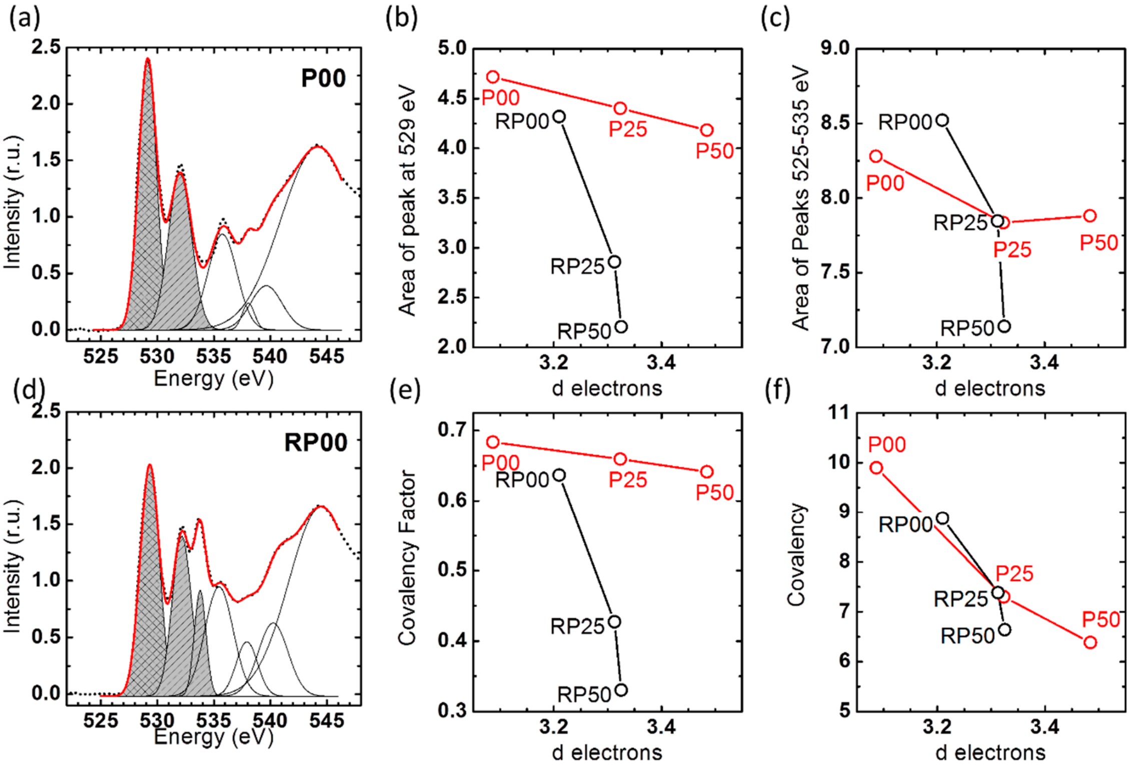

Figure S8. Determination of the covalency factor by the procedure used herein compared to the method by Suntivich et al. [6]. Here, we used only the pre-peak at lowest energy (\ \ \ shading in **a**,**d**) and divided it by the number of 3*d* holes calculated using the Mn valence. On the other hand, Suntivich et al. used the area of all peaks assigned to e_g and t_{2g} (/// shading in **a**,**d**) and divided it by (e_g + 0.25 t_{2g}) occupancy. The trends in (**b**) total O-K peak area of the peak at 529 eV are shown side by side to (**c**) the area of all peaks in the O-K pre-edge between 525 and 535 eV as a function of Mn *d*-electron occupation. The resulting covalency factors are plotted in (**e**,**f**), respectively. Please refer to the text for further discussion.

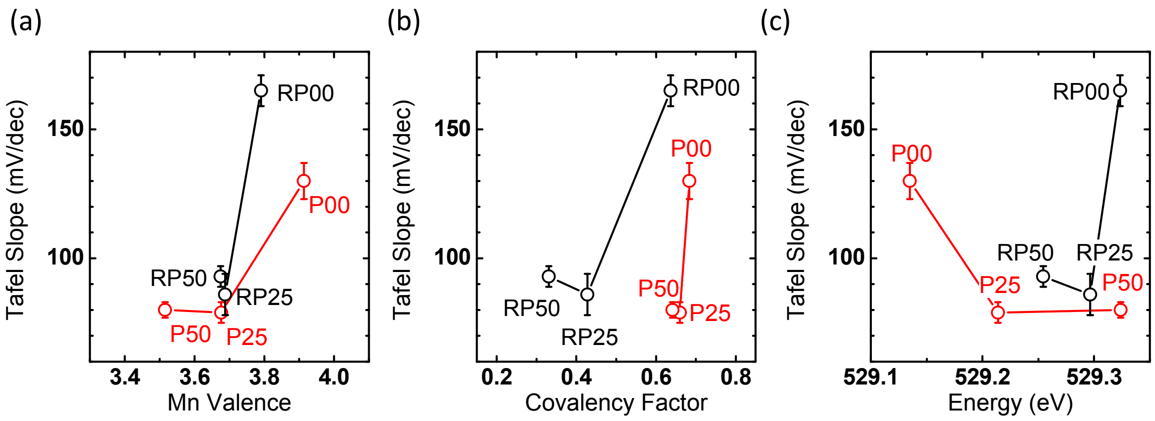

Figure S9. Trend of the Tafel slope with (**a**) Mn valence; (**b**) covalency factor and (**c**) energy position of the e_g pre-edge in O-K XAS.

References

1. Sheng, W.; Gasteiger, H.A.; Shao-Horn, Y. Hydrogen Oxidation and Evolution Reaction Kinetics on Platinum: Acid vs. Alkaline Electrolytes. *J. Electrochem. Soc.* **2010**, *157*, B1529–B1536.
2. Suntivich, J.; May, K.J.; Gasteiger, H.A.; Goodenough, J.B.; Shao-Horn, Y. A Perovskite Oxide Optimized for Oxygen Evolution Catalysis from Molecular Orbital Principles. *Science* **2011**, *334*, 1383–1385.
3. Risch, M.; Stoerzinger, K.A.; Maruyama, S.; Hong, W.T.; Takeuchi, I.; Shao-Horn, Y. $La_{0.8}Sr_{0.2}MnO_{3-\delta}$ Decorated with $Ba_{0.5}Sr_{0.5}Co_{0.8}Fe_{0.2}O_{3-\delta}$: A Bifunctional Surface for Oxygen Electrocatalysis with Enhanced Stability and Activity. *J. Am. Chem. Soc.* **2014**, *136*, 5229–5232.
4. Grimaud, A.; Carlton, C.E.; Risch, M.; Hong, W.T.; May, K.J.; Shao-Horn, Y. Oxygen Evolution Activity and Stability of $Ba_6Mn_5O_{16}$, $Sr_4Mn_2CoO_9$, and $Sr_6Co_5O_{15}$: The Influence of Transition Metal Coordination. *J. Phys. Chem. C* **2013**, *117*, 25926–25932.
5. Mildner, S.; Hoffmann, J.; Blöchl, P.E.; Techert, S.; Jooss, C. Temperature- and doping-dependent optical absorption in the small-polaron system $Pr_{1-x}Ca_xMnO_3$. *Phys. Rev. B* **2015**, *92*, 035145.
6. Suntivich, J.; Hong, W.T.; Lee, Y.-L.; Rondinelli, J.M.; Yang, W.; Goodenough, J.B.; Dabrowski, B.; Freeland, J.W.; Shao-Horn, Y. Estimating Hybridization of Transition Metal and Oxygen States in Perovskites from O K-edge X-ray Absorption Spectroscopy. *J. Phys. Chem. C* **2014**, *118*, 1856–1863.
